# Comparative Study of Hydrogen Reduction of Bauxite Residue-Calcium Sintered and Self-Hardened Pellets Followed by Magnetic Separation for Iron Recovery

**DOI:** 10.1007/s42461-023-00882-3

**Published:** 2023-11-23

**Authors:** Manish Kumar Kar, Ahmad Hassanzadeh, Casper van der Eijk, Jafar Safarian

**Affiliations:** 1 Department of Materials Science and Engineering, Norwegian University of Science and Technology, Alfred Getz Vei 2, 7034 Trondheim, Norway; 2 Department of Geoscience and Petroleum, Faculty of Engineering, Norwegian University of Science and Technology, Andersens Veg 15a, 7031 Trondheim, Norway; 3 SINTEF Industry, Torgarden, Trondheim, Norway

**Keywords:** Hydrogen reduction, Bauxite residue, Sintered pellet, Self-hardened pellet, Thermogravimetry, Mayenite

## Abstract

To minimize the carbon footprint in the industrial valourization of bauxite residue, hydrogen was used as a reducing agent. The current study experimentally investigated hydrogen reduction of bauxite residue-CaO sintered and self-hardened pellets at 1000 °C, along with magnetic separation of these reduced pellets for iron recovery. Calcium was introduced to bauxite residue to form leachable calcium aluminate phases with the existing alumina in bauxite residue. This involved the addition of either CaCO_3_ or a mixture of CaO and CaCO_3_ while maintaining the fixed Ca content during pelletization. The former underwent sintering at 1150 °C, while the latter was self-hardened through the cementing effect of CaO in exposure to moisture and air. Both types of pellets were reduced in a thermogravimetry furnace at an elevated temperature under similar conditions. The pellets were characterized by the X-ray diffraction (XRD) method and scanning electron microscope (SEM) coupled with energy dispersive spectroscopy (EDS), and their physical and mechanical properties were measured via standard techniques. During hydrogen reduction, a negligible amount of gehlenite (Ca_2_Al_2_SiO_7_) was formed in the self-hardened pellets, while this phase dominated in the sintered pellets. Alumina in the bauxite residue converted to mayenite phases during reduction in both the pellet types; however, reduced self-hardened pellets had a higher amount of alumina containing mayenite leachable phase. The two pellets showed similar reduction behaviour, while different chemical, physical, and mechanical properties were observed. The magnetic properties of milled reduced pellets were examined through a Davis Tube magnetic separator in a wet environment under a constant magnetic field of 800 G. Higher iron recovery was observed for the self-hardened reduced (41%) pellets than for the sintered pellets (27%).

## Introduction

As the modernization of society is growing, the demand for aluminium has increased over time. Bauxite is the major raw material for the production of alumina. Most alumina is produced around the world through the Bayer process, which generates a waste known as red mud or bauxite residue (BR) in dewatered form [1]. However, the generation of BR has serious environmental challenges due to its properties and storage difficulties [1]. Many researchers have tried to find an efficient way of the utilization of BR because it can be a potential secondary source for the metals like Fe, Al, Ti, Ca, and rare earth elements. However, utilization of BR is still impenetrable mainly due to economic reasons and running efficient processes. Therefore, it is important to find a different technology for using BR to eliminate the environmental problem [2]. Carbon as a reductant is still used as dominating reduction technology globally; at the same time, the metal industry is trying to shift towards global decarburization. Hydrogen can be a suitable alternative as a reductant for the primary metal production and metal recycling processes [3]. As the environmental greenhouse gas concern increases, green reduction will be a promising technology for iron oxide reduction in BR.

Most of the iron production in the world is made through the blast furnace and basic oxygen furnace (BF/BOF) route, which needs coking coal both as a reductant and heat source. As the shortage of coking coal and the environmental climate concern increase, hydrogen can most likely be the future reductant for the steel industry [4]. Globalization increases with time, and for future economic growth, industry has to adopt low carbon technologies [5, 6]. There is some major difference underlined between hydrogen reduction and carbothermic or carbon monoxide reduction from a metallurgical point of view. Iron oxide reduction with hydrogen is endothermic, however, the reduction kinetics is faster; therefore, the industrial operation must be different compared to conventional carbothermic reduction [7]. The reduction of iron oxide by hydrogen goes through either two or three steps depending upon the applied reduction temperature. At a temperature higher than 570 °C, hematite (Fe_2_O_3_) transforms to magnetite (Fe_3_O_4_) and wustite (FeO) and finally to metallic iron but at temperatures below 570 °C, hematite to magnetite first, and then directly to metallic iron because wustite is not stable below 570 °C [7, 8].

Limited research works have been carried out in literature on the hydrogen reduction of BR and bauxite ore. Low temperature hydrogen reduction of BR has been studied for the conversion of Fe_2_O_3_ to Fe_3_O_4_ and formation of leachable sodium aluminate phase [9]. A group of researchers found the maximum conversion of hematite to magnetite around 96% with 20 wt% NaOH addition and reduction with 5 vol% H_2_ for 120 min at 500 °C. Further, hydrogen reduction of bauxite ore was studied for the iron separation prior the ore smelting [2]. It was found that the reduction of hematite to iron starts below 560 °C and the rate and extent of reduction improved with increasing temperature; however, formation of hercynite (FeAl_2_O_4_) retards the complete reduction above 760 °C. The addition of sufficient Ca to BR avoids the hercynite formation during the reduction step. Another study of hydrogen reduction, where BR and calcite sintered pellets, were carried out using hydrogen at 1000 °C, 1100 °C, and 1200 °C. They reported that the reduction rate was faster at 1000 °C compared to 1100 °C and 1200 °C, which was due to the porosity loss at higher temperatures and the reduction mechanism changed to diffusion-controlled step. For the reduced sample at 1000 °C, most of iron oxide complexes were reduced to the metallic iron and alumina recovery raised up to 87% by alkali leaching of calcium aluminate phase (Ca_12_Al_14_O_33_) [10].

The main component of BR is iron which is in the form of mainly hematite. Although iron extraction using physical, chemical, and physicochemical processes in laboratory scale has been addressed extensively in the literature, there is very little information available regarding processibility of hydrogen-reduced BR. In this regard, Li et al. [11] applied a high gradient superconducting magnetic separation (HGSMS) where mass recovery of only about 10% was reported. Rai et al. [12] utilized different reductants including H_2_ where maximum Fe_2_O_3_ content could be reached was 60% under single/multiple stages of magnetic separation with intensities up to 15,000 G. In another study, Kapelari et al. [13] employed water leaching followed by dry magnetic separation led to reaching 38.5 wt% Fe content, whereas a wet magnetic separation resulted in 31.8 wt% Fe. As seen, ultimate Fe percentage and its recovery are highly dependent on the applied process. It is worth noting that, for a profound perception of each process, one should pay attention to the ore properties, particle size, iron oxide contents in the feed, and reduction procedures for each case individually.

The present work focuses on hydrogen reduction behaviour of BR calcium (sintered and self-hardened) pellets, phase formations during reduction process, properties of reduced pellets, and iron recovery by wet magnetic separation of two types of pellets with similar reduction and magnetic separation conditions. Addition of calcium-containing raw material initiates the formation of the calcium aluminate phase during reduction. This calcium aluminate phase is characterized by its alkali leachability properties, which facilities the subsequent alumina recovery process from the reduced pellets. The placement of CaO in CaCO_3_ gives strengthening to the pellets so that we can omit the application of high temperature sintering process before the hydrogen reduction.

CaCO_3_ is partly substituted by CaO in order to make self-hardening pellets that do not need to be sintered before a reduction in hydrogen, thereby reducing the cost of the process. The effect of CaO addition on the pellet’s strength and the phase formation also is studied in detail.

## Materials and Methods

### Materials

Bauxite residue (BR), limestone (CaCO_3_), quick lime (CaO), and water were used for pelletization. BR was supplied from the Mytilineos metallurgy business unit S.A. (previously known as aluminium of Greece). The limestone was provided by VUGIUKLI SA, Greece, and quick lime was supplied from the NorFraKalk. The materials were dried in an oven overnight at 80 °C and deagglometered and sieved below 500 µm.

### Pelletization Process

BR to limestone (61.01:38.9) wt% and BR to limestone to quicklime (63.1:32.3:4.5) wt% were used for pelletization of sinter pellets and self-hardened pellets, respectively. These two types of mixtures were blended in a tubular mixture for 30 min for homogenization of the materials. However, in both mixtures, the BR to calcium ratio remained constant. Chemical compositions of BR, limestone, quick lime, and produced pellets are presented in Table 1.

**Table 1:** Chemical composition of BR, limestone, quicklime, and respective pellets (wt%)

Materials/oxides	Al_2_O_3_	CaO	Fe_2_O_3_	K_2_O	MnO	MgO	Na_2_O	P_2_O_5_	SO_3_	SiO_2_	TiO_2_	L.O.I
BR	22	8.8	40.71	0.09	0.08	0.23	3.1	0.11	0.95	7.1	5	12.4
Limestone	0.9	52.7	0.15	0.12	DL	0.95	DL	0.01	0.06	2.07	0.03	42.6
Quick lime	0.24	96.7	0.08	DL	DL	0.64	DL	0.02	DL	0.46	0.02	0.5
Sintered reduced	23.6	36.0	21.5(Fe)	0.09	0.05	0.9	2.2	0.1	0.8	9.7	4.4	0.22
Self-hardened reduced	18.7	39.3	22.5(Fe)	0.05	0.03	0.61	2.5	0.11	0.9	7.1	4.9	2.9

*DL* detection limit

*L.O.I.* loss of ignition at 1000 °C for 1 h as per ISO 17025

The mixed materials were pelletized in a disk pelletizer with 45º rotation angle and rotational speed of 25 rpm via the addition of water. The water addition was the same for both types of pellets which was about 10 wt%. During pelletization, as the disc undergoes rotation, mixture of bauxite residue and calcium oxide/carbonate is charged at the bottom of the disc. On contact with water from the sprayer initiates the nucleation phase. The rolling action promotes formation of small nuclei at the lower section of the pelletizer. The added mixture agglomerates on the surface of the nuclei, and it begins to increase the pellet size. Mixture addition and water spraying continues until the pellets size exceeded above 5 mm, and they are manually collected from the rotating disc. Then, the pellets are sieved and sorted within a size range of 5 to 10 mm; smaller particles are fed back to the rotating disc.

First group of green pellets were dried overnight at 80 ± 5 °C, and the dried pellets were sintered at about 1150 ± 10 °C in a muffle furnace for 120 min and furnace cooled down. The second one was self-hardened by keeping in air. For both pellets, size was about 6 to 10 mm. In this work, we used two nomenclatures for the two pellets: First is “sintered pellet”, and second one is “self-hardened pellet”.

### Characterization

The mineralogical phase analysis of materials was performed by the X-ray Diffraction (XRD) using Bruker D8 A25 DaVinciTM (Karlsruhe, Germany) with the CuKα radiation in 2θ range from 15 to 75° diffraction wavelength with a step size of 0.03°. The chemical composition of the material was determined by the X-ray Fluorescence (XRF) supplied by the Thermo Fisher (Degerfors lab AB, Sweden). Microstructural analysis of the treated material was carried out by a scanning electron microscope (SEM) supplied by the Zeiss ultra 55LE (Carl Zeiss. Jena, Germany). The elemental distribution was assessed using elemental mapping via energy dispersive spectroscopy (EDS), Bruker AXS, microanalysis GmbH (Berlin, Germany).

To determine the strength of the pellets, a compact hydraulic press was utilized. Three measurements for each pellet were executed, and the average value was considered for the calculation of the breaking load. For the tumbler and abrasion test, we used pellets of size greater than 7-mm diameter in a mini-tumbler test unit in which (ISO 3271, 3^rd^ edition standard [14]) the tumble is re-scaled to a smaller size. As the quantity of the materials is very much as per international standard for tumbling test, which is not convenient for conducting a lab scale experimental work, it was rescaled to the smaller size. The dimension of rescale tumbler was 200-mm inner diameter and 12-mm length including 4 L with the height of 6 mm. Abrasion index (AI) calculated based on the weight fraction of fines below 500 µm was divided to the total weight pellets. Tumble index (TI) was calculated as the weight fraction above 6.3-mm pellets divided by the total weight of the pellets.

### Hydrogen Reduction of Pellets

**Fig. 1:**
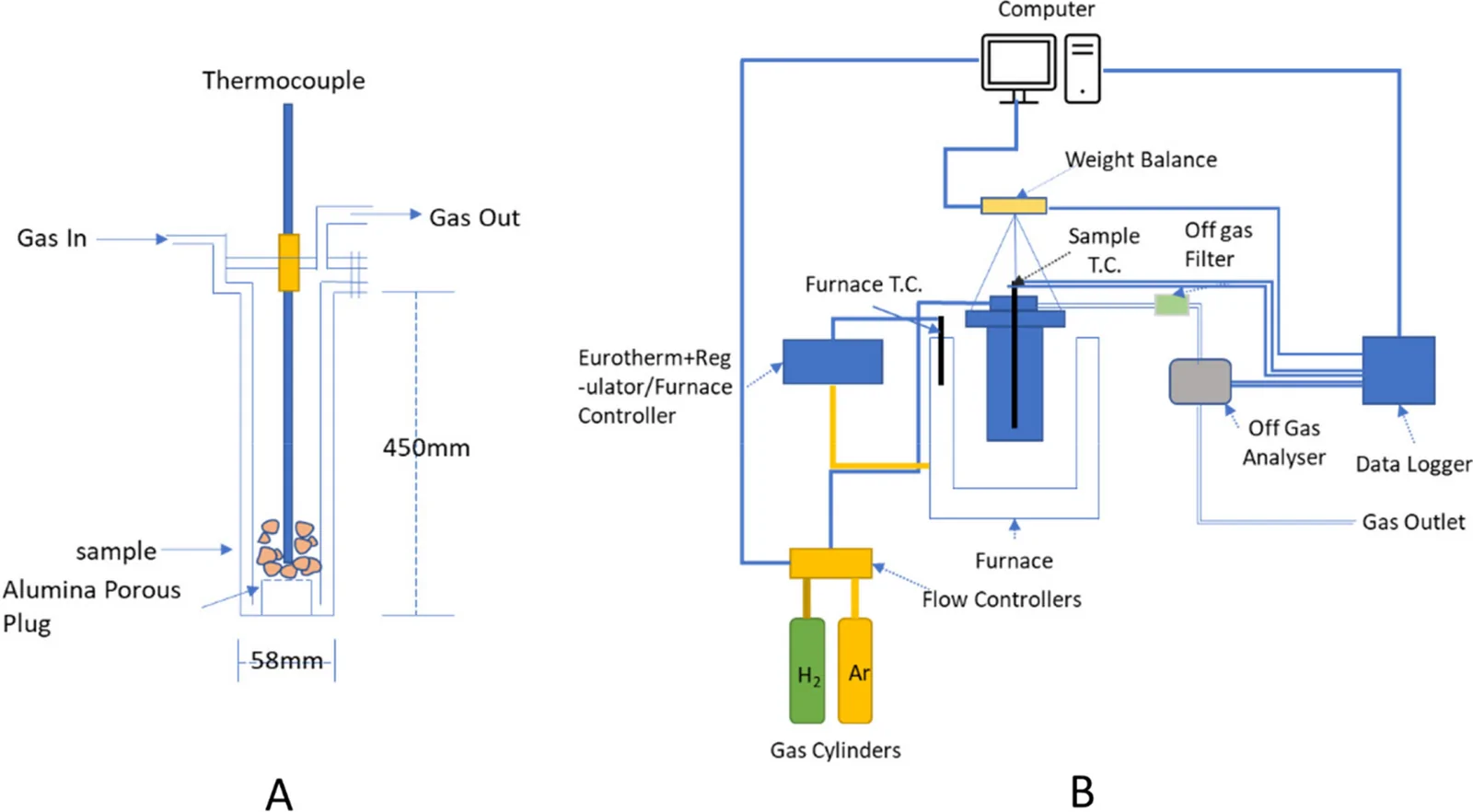
**A** Schematic view of crucible and **B** the overall furnace setup

Direct reduction of both type of the pellets by hydrogen gas was carried out in a thermogravimetry furnace. The setup of the furnace is shown in Fig. 1: the crucible schematic (Fig. 1A) and the whole furnace setup (Fig. 1B). A thermocouple was inserted in the middle of the crucible to measure the temperature of the sample. The pellets were heated at the rate of 10 °C/min to 1000 ± 10 °C with argon purging 1NL/min, and after the reduction step, sample cooling was implemented with the same flow rate of argon. The optimisation of reduction temperature, time, and hydrogen flow rate was studied previously [15]. In the reduction step, the argon purging was changed to only hydrogen with a flow rate of 4 NL/min for about 90 min. Two thermocouples were used; one is at the furnace wall, and another one inside the sample bed. Based on the two-thermocouple reading, targeted temperature was set.

### Davis Tube Test Procedure

To analyse the feasibility of magnetic separatorability for both pellet types, they were subjected to the Davis Tube tests (Dings Magnetic Separator Co., USA) under a constant magnetic field of 800 G. Both samples were crushed using a jaw crusher (BB100, Retsch®, Germany) and subsequently sieved using a dry screening machine (RX-29H&B, ROT-AP®, W.S. Tyler, USA) to obtain 20 g of each sample at the size fraction of 100–200 µm for the magnetic tests. This optimum particle size fraction was obtained through previous test works [16].

Before each test, the strike was regulated to have 60 strokes/min, and the water flow rate was stabilized at 1 L/min. The prepared 20-g sample was added using a peristaltic pump with the speed of 120 rpm, and the non-magnetic sample was gathered from the bottom of the tube, while the magnetic fraction (concentrate) was collected by disconnecting the magnetic field at the end of the experiment. The magnetic and non-magnetic products were filtered, dried for 1 h at 60 °C, weighed, and analysed using the XRF method. The recovery was calculated using the well-known equation as $R=\left(\frac{C}{F}\right)\times \left(\frac{c}{f}\right)*100$, where the *R*(%) is the recovery, *C*(g) and *F*(g) are the weight of magnetic and non-magnetic products, and *c*(%) and *f*(%) denote the grade of Fe in the magnetic product and in the feed sample, respectively.

## Results and Discussions

### Results

#### Phase Analyses

**Fig. 2:**
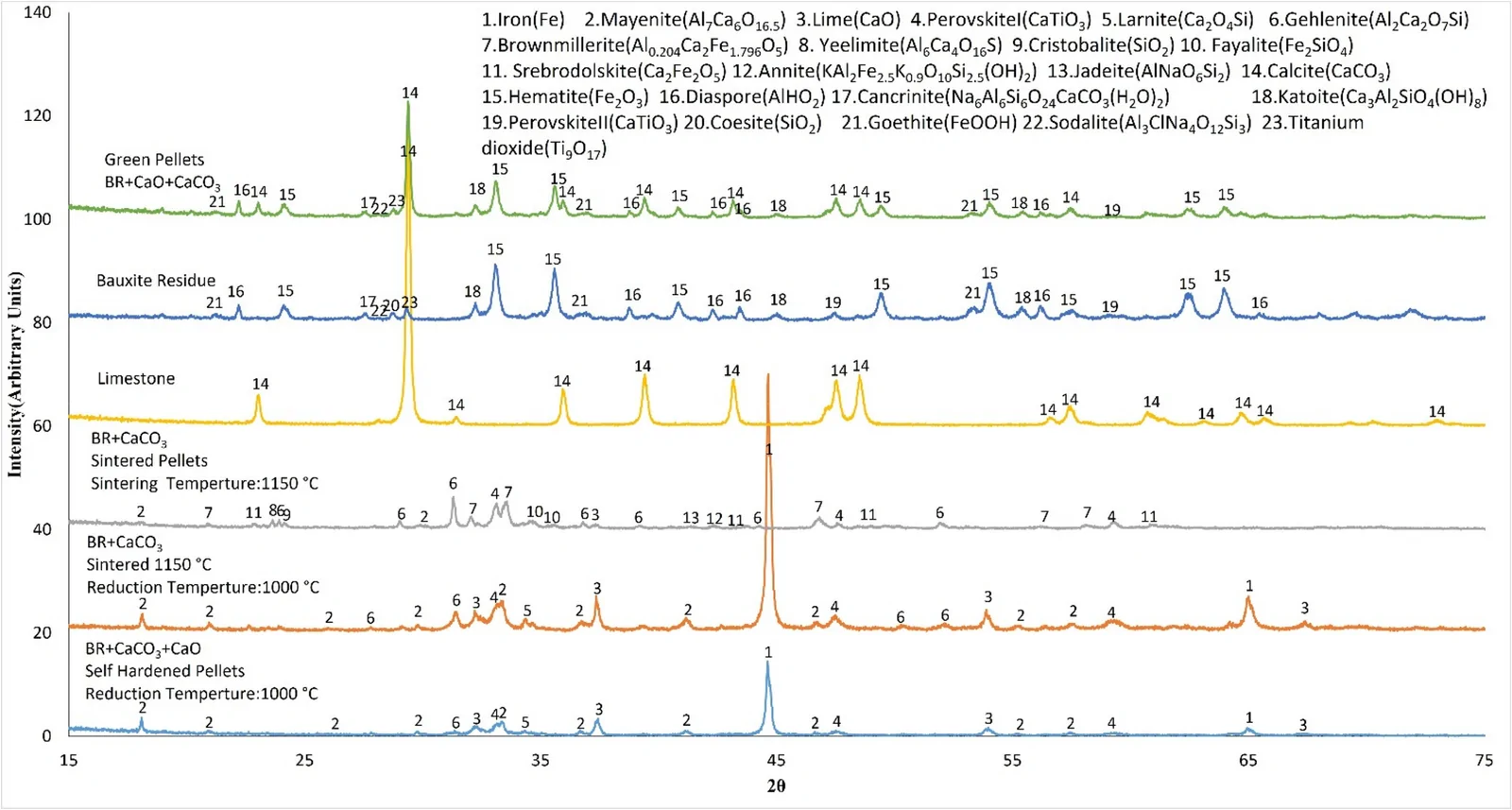
XRD diffractograms of BR, limestone, green self-hardened pellet, sintered pellet, reduced self-hardened pellet, and reduced-sintered pellet

As the XRD patterns demonstrated in Fig. 2, iron is present in the BR mostly as hematite (Fe_2_O_3_) and goethite (FeO(OH)). It can be seen that the metallic iron in the reduced sintered pellets has a higher magnitude compared to that in the reduced self-hardened pellets. Aluminium is present in many phases such as diaspore (AlO(OH)), cancrinite (C_0.76_H_1.7_Al_3_Ca_0.75_Na_3_O_14.4_Si_3_), katoite (AlCa_3_H_9.7_O_12_Si_0.69_), and sodalite (Al_3_ClNa_4_O_12_Si_3_). Other than these phases, BR also contains perovskite (CaTiO_3_), coesite (SiO_2_), anatase (TiO_2_), and calcite (CaCO_3_). The identified codes of the bauxite residue is given in detail in Appendix A. Limestone is mainly composed of calcite (CaCO_3_), which its XRD spectra and XRD analyses can be found in Fig. 2 Table 1, respectively.

It can be seen in Fig. 2 that in the reduced self-hardened pellets, the intensity of gehlenite (Ca_2_Al_2_SiO_7_) spectrum is lower compared to that in the reduced sintered pellet. In the sintered pellets, iron presents in the form of brownmillerite (Ca_2_(Fe,Al)_2_O_5_), srebrodolskite (Ca_2_Fe_2_O_5_), and fayalite (Fe_2_SiO_4_); however, major fraction present is in the form of brownmillerite. During high temperature, hydrogen reduction alumina in the BR reacts with CaO and forms mayenite, which is highly alkali leachable phase from previous study [10]. Reduced self-hardened pellet has higher intensity of mayenite (Ca_12_Al_14_O_33_) compared to that in the reduced sintered pellet. The calcium oxide (CaO) shows a significantly higher intensity spectrum in the reduced sintered pellet compared to the self-hardened reduced pellet. In the green self-hardened pellets, all the calcium present is in calcite (CaCO_3_) form.

#### Microstructural Analysis

**Fig. 3:**
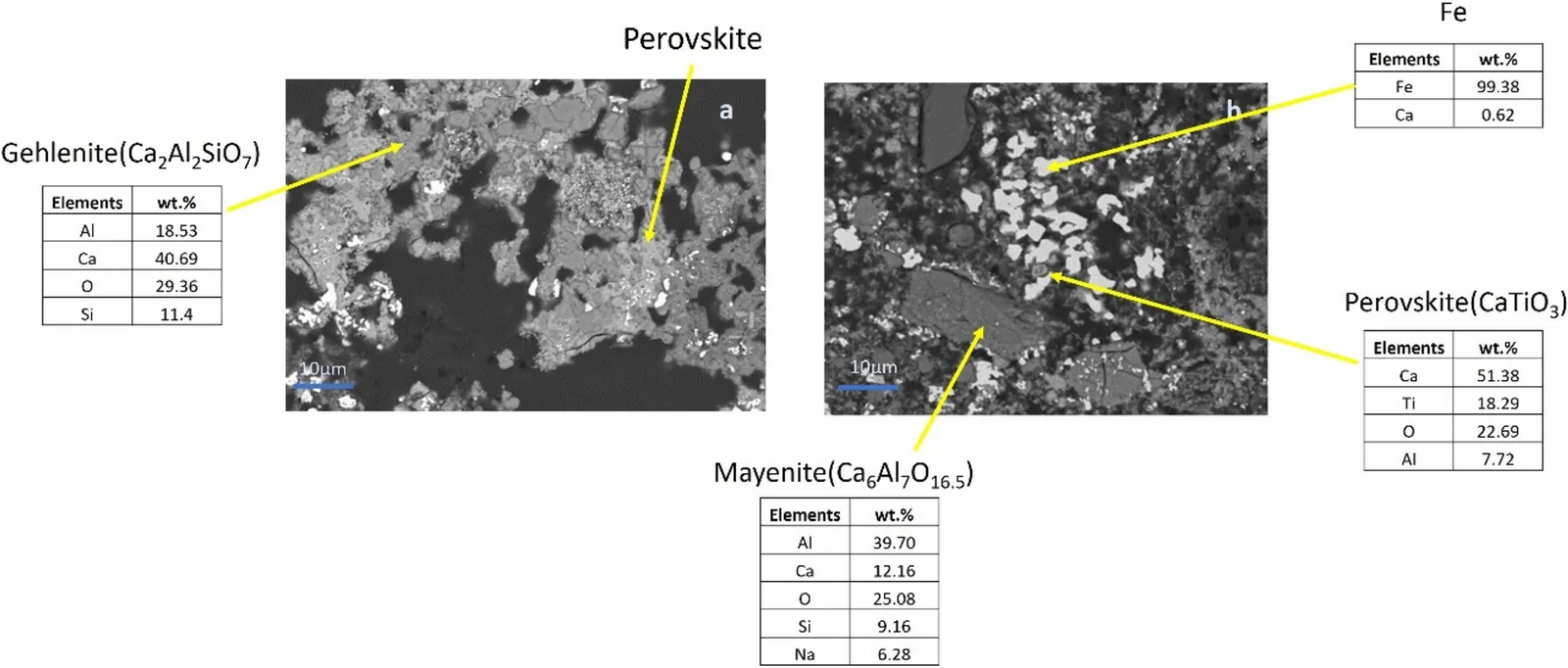
SEM images and atomic analyses of **a** the reduced sintered and **b** reduced self-hardened pellets

Figure 3 displays the SEM results and dot analyses of the identified phases. As per the SEM images, there is more clustering of metallic iron particle in reduce self-hardened pellets in comparison with the reduced sintered pellets. In the reduced sintered pellet, the iron particle size is finer and distributed all over the matrix shown in Fig. 3a. CaTiO_3_ exists in both reduced pellets, and Ca_2_Al_2_SiO_7_ presents higher amount in reduced sintered pellets (Fig. 3a).

**Fig. 4:**
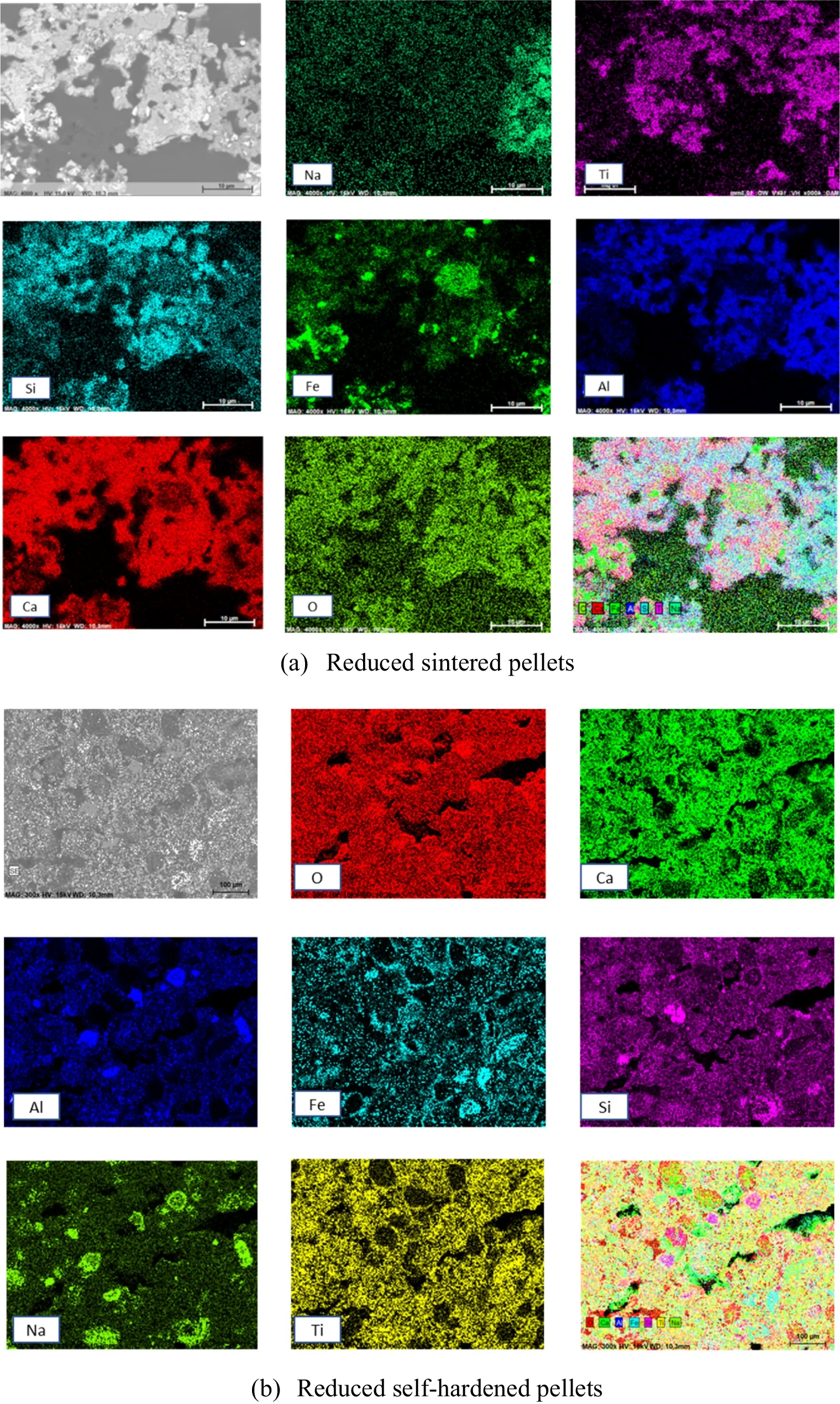
Elemental mapping of **a** reduced sintered pellets and **b** reduced self-hardened pellets

Figure 4a and b show the EDS elemental mapping for the two reduced pellets. In the reduced sintered pellet, Ca, Al, and Si are associated with O, which is most likely the gehlenite phase, but there is not an intensive association in the reduce self-hardened pellet. In both pellets, Ca, Ti, and O are present in the form of CaTiO_3_ according to the X-ray mapping results, which is well correlated with the XRD patterns (Fig. 2). In mayenite, along with Al, Ca, and O, some fraction of Si and Na is also detected, likely originated from the surrounding signals.

**Fig. 5:**
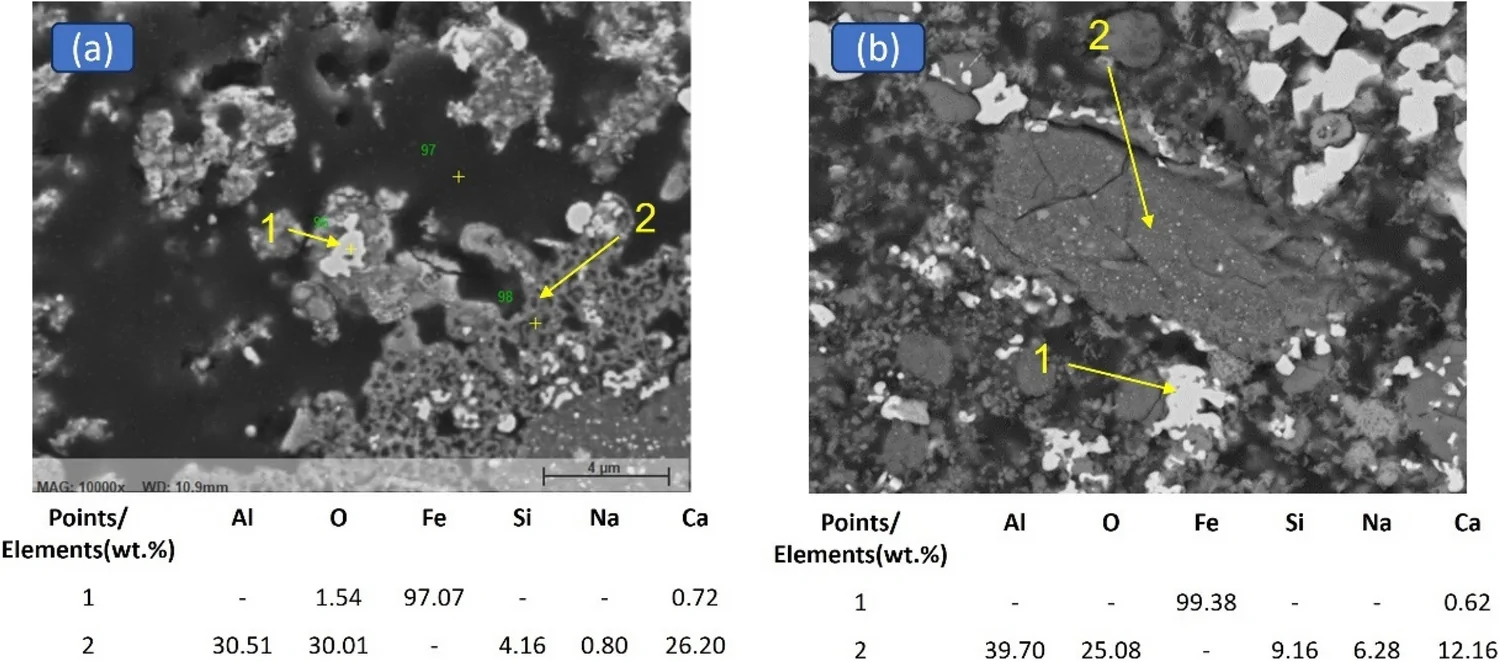
Elemental point analysis of iron and mayenite of **a** reduced sintered pellet and **b** reduced self-hardened pellet

The EDS point analysis of reduced sintered and recued self-harden pellets is shown in Fig. 5a and b, respectively. Iron and mayenite elemental analysis are presented for these two pellets. It is shown that in mayenite along with Al, Ca, and O, some fractions of Si and Na are present.

#### Mechanical Properties

Based on the visual observation, it was found that the pellet sintered at 1150 °C for 120 min had better strength compared to the self-hardened pellet. According to the results shown in Table 2, the measured tumble index is low for the self-hardened pellet compared to the sintered pellet. Good strength of pellets can be evaluated considering the abrasion index below 6% and tumble index above 92% [14]. The tumble index of 55.2% for self-hardened pellets and 88.3% for the sintered pellets were obtained from the experimental tests. Lower abrasion index indicates less degradation and dust formation from the pellets in handling and in the process. It can be seen that the self-hardened pellets have higher abrasion index of 37.11%, which shows that this pellet is much more susceptible to abrasion. In addition, the breaking load for sintered pellets is one order magnitude higher than the self-hardened pellets.

**Table 2:** Comparison of mechanical properties of two different pellets

	Self-hardened pellets	Sintered pellet
Breaking load (kN) (average of 3 pellets)	0.023	0.29
Tumbling index (%)	55.22	88.33
Abrasion index (%)	37.11	4.89

The measured density for the reduced sintered pellet was around 3.55 g/cm^3^, and it was around 3.57 g/cm^3^ for the reduced self-hardened pellet. The measured porosity of the sintered pellet was around 54.99 vol.%, while for the self-hardened pellet, this value was around 57.2 vol.%.

#### Weight Loss During Hydrogen Reduction

**Fig. 6:**
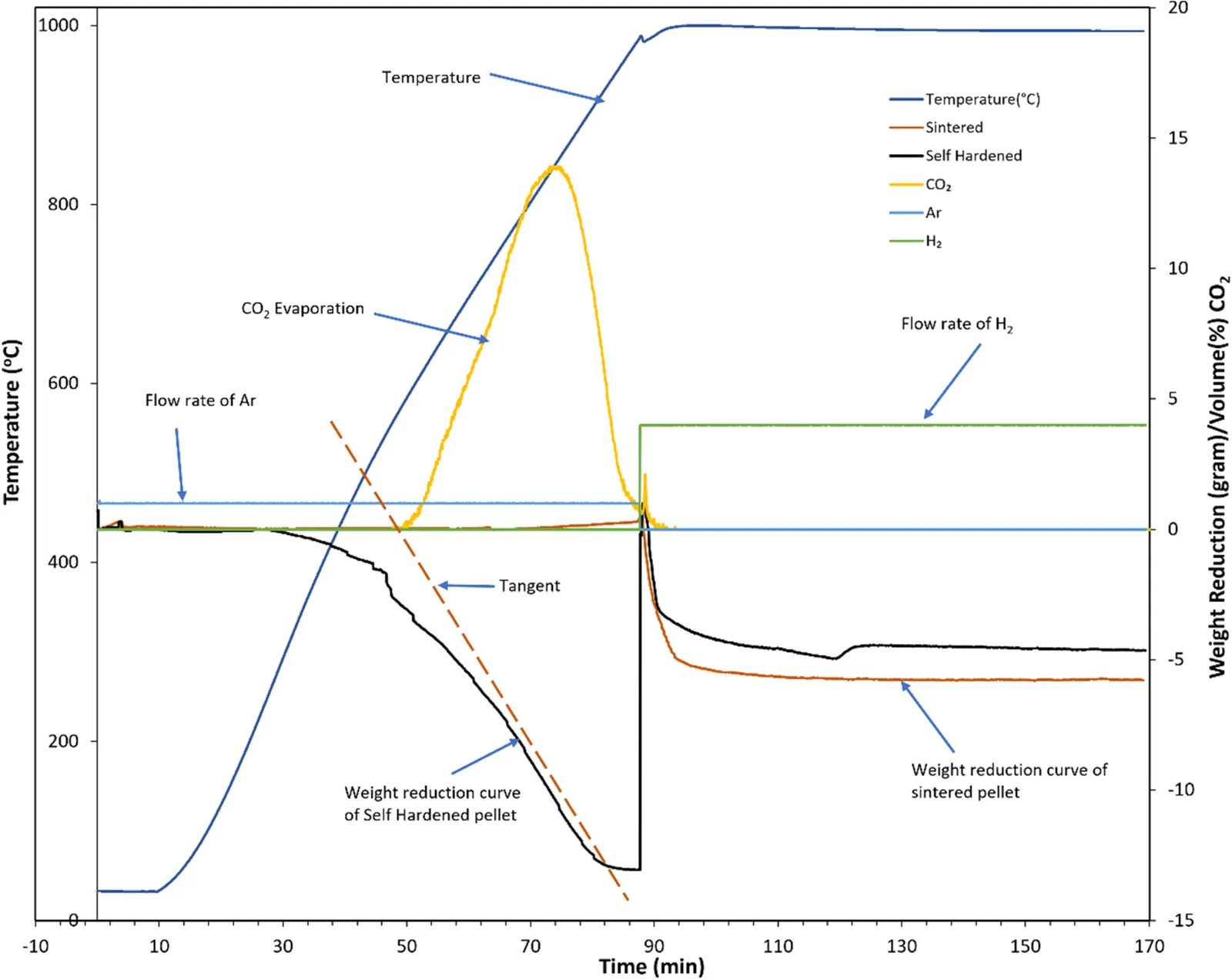
Weight reduction vs time graphs of sintered and self-hardened pellets

Figure 6 shows the weight changes, heating temperature, CO_2_ evaporation, and gases flow rate with respect to the time for two types of pellets. As seen, there is no weight loss for the sintered pellet sample during the heating step in Ar atmosphere, but in the self-hardened pellets, there is weight loss, which is around 25.2 wt% of the sample. Initially, there is very small weight loss of the self-hardened pellets, but after about 40 min of heating and when temperature is around 550 °C, there is drastic weight loss, which is shown as onset temperature in Fig. 6. Drawing tangents over the mass reduction curve for this pellet gives this temperature. The calcite decomposition starts at around 550 °C and highest rate of decomposition at around 830 °C, which is shown in Fig. 6. As per theoretical calculation 887 °C (as per HSC chemistry 9), however, in actual heating, it starts at lower temperature which may be due to the lowering equilibrium partial pressure as heating carried out in presence of argon. In the reduction regime, when hydrogen is introduced, the mass loss in reduce sintered pellets is around 5.71 g and reduces self-hardened pellets around 4.713 g, which is near to theoretical mass loss for full reduction of iron oxide (Sect. 4.3).

#### Davis Tube Test Results

Table 3 represents the iron metallurgical responses obtained though Davis Tube tests for both sample types. As can be evidently observed, under identical operating conditions, higher iron recovery is captured for the self-hardened reduced sample compared to the sintered reduced one. The main reason for this phenomenon is attributed to a favourable clustering of iron-bearing component in the reduced self-hardened pellets as clearly shown in Fig. 3. This indeed led to higher mass recovery a.k.a yield (i.e., C/F value) for the respective sample. However, the enrichment ratio as another key metallurgical response (c/f) for the physical beneficiation process showed a poor improvement in iron grade for both samples, i.e., 1.07 and 1.12, for self-hardened reduced and sintered reduced one, respectively. This low improvement in iron grade can be mainly related to the complex mineralogy of both samples and metallic iron dissemination throughout the sample’s matrixes. Further grinding the samples to below 5–10 µm and applying physiochemically-based separation processes such as flocculation-flotation method can be potentially heighten this ratio which is the matter of future studies.

**Table 3:** Iron recovery and enrichment ratio acquired via wet magnetic separation tests

Sample type	Self-hardened reduced	Sintered reduced
Recovery (%)	41	27
Enrichment ratio	1.07	1.12

### Discussions

The obtained results presented above are discussed as follows.

#### Mechanical Properties of Oxide Pellets

**Fig. 7:**
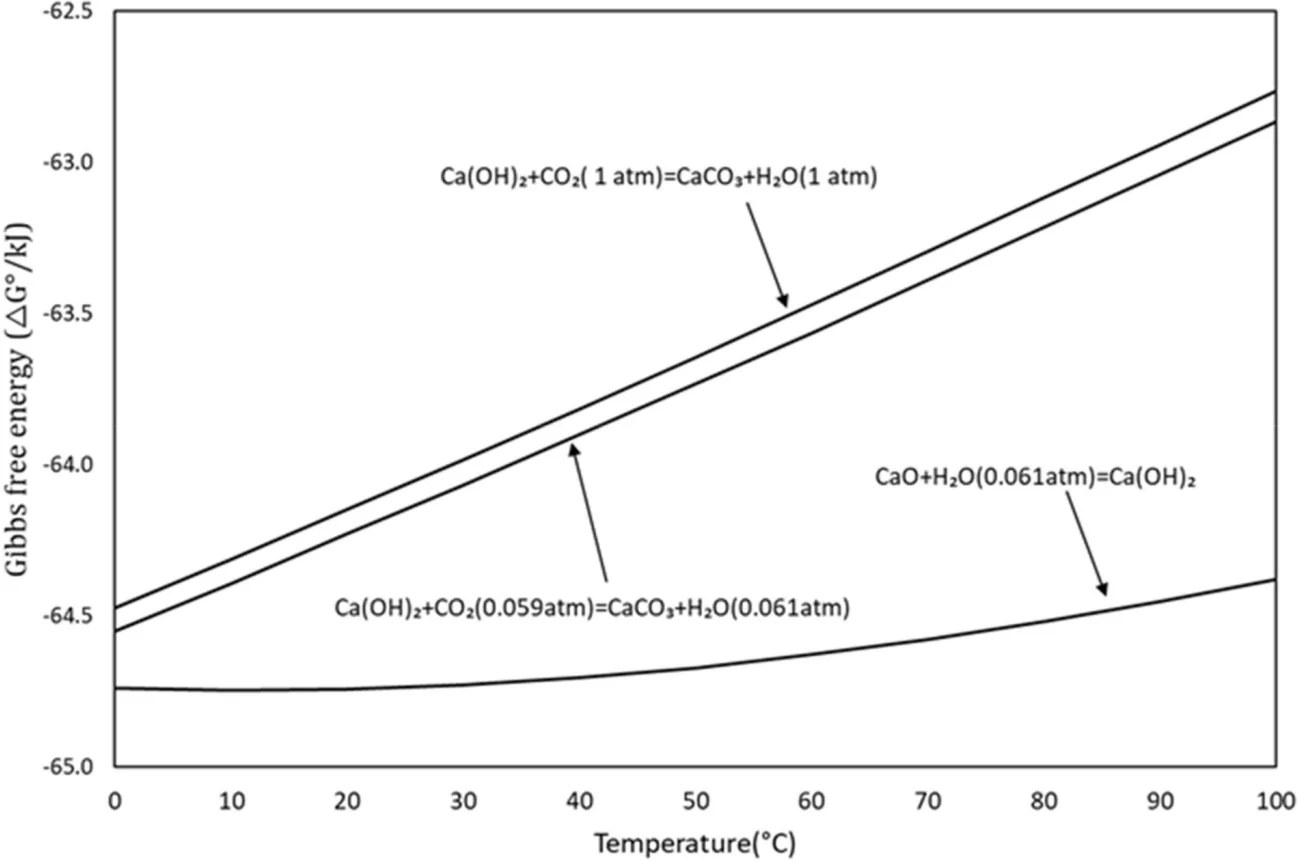
Gibbs free energy changes for the formation of Ca(OH)_2_(1atm) and CaCO_3_ (0.06 atm CO_2_ and 0.061 atm H_2_O), calculated by HSC chemistry 9

There is a significant difference between the strength of sintered and self-hardened pellets, and the sintered oxide pellet showed better performance (Sect. 3.1.3). Strength of sintered pellets is due to high temperature heating which results in partial fusion and agglomeration of oxides in matrix; however, strength of the dried self-hardened pellets comes from the chemical reaction of quick lime. Quick lime has excellent cementitious property[17] which gives strength to the self-hardened pellets. When quick lime reacts with moisture, it forms Ca(OH)_2_, and the hardening property of quick lime comes when Ca(OH)_2_ reacts with CO_2_ in air to from CaCO_3_ which is in accord with the XRD results (Fig. 1). The detection of no CaO and Ca(OH)_2_ in the self-hardened pellet can be taken as a confirmation of the completion of reaction. In order to evaluate the changes, the Gibbs free energy change with temperature for the main reactions were calculated and are plotted in Fig. 7. It can be seen that the standard Gibbs energy of formation (△G^0^) of Ca(OH)_2_ from CaO and H_2_O and then the formation of CaCO_3_ from Ca(OH)_2_ at 25 °C are both negative and hence the reactions occurs from thermodynamic point of view. As the CO_2_ atmospheric pressure in air is low (about 0.061 atm) and the H_2_O atmospheric pressure is also low (0.059 atm), the changes in Gibbs free energy of formation(△G) of CaCO_3_ at 25 °C were calculated regarding these low pressures, and the negative values (Fig. 7) indicate that the reaction can proceed in pellets in exposure to air. Formation of Ca(OH)_2_ and CaCO_3_ are negative at room temperature, and free energy becomes less negative with rise in temperature.

With this specified CaO and CaCO_3_ ratio in self-hardened pellets, the porosity of self-hardened pellets is higher than the sintered pellets due to oxide phases agglomeration during high temperature sintering. In sintering, CaCO_3_ decomposition results in increase in porosity; however, at the same time, surface or partial fusion of particles occurs which results in decrease porosity of the pellets. In addition, for making the self-hardened pellets, CaCO_3_ and CaO were added, and the chemical and thermal behaviour of these species affect the pellet porosity during the heating and reduction steps. Here, both hydration of quick lime and carbonation of Ca(OH)_2_ to CaCO_3_ have volume expansion [18], which results in increases in porosity.

#### Evolution of Phases During Sintering and Reduction

During the reduction of the self-hardened pellets, there is very little formation of the gehlenite (Ca_2_Al_2_SiO_7_) phase as compared to reduced sintered pellets. For the sintered pellet, during sintering, the free calcium oxide (formed via CaCO_3_ decomposition) reacts with alumina and silica in the BR to form Ca_2_Al_2_SiO_7_. Moreover, CaO reacts with alumina and iron oxides to form the brownmillerite (Ca_2_(Fe,Al)_2_O_5_) phase. The formation of these phases is confirmed with regard to the sintered oxide pellet XRD spectrum (Fig. 2), and microstructural analysis results presented above. According to XRD result of the corresponding reduced sample, the formed brownmillerite (Ca_2_(Fe,Al)_2_O_5_) phase in the reduction step is converted to metallic iron, mayenite (Ca_12_Al_14_O_33_), and CaO, which is presented via Eq. (1). The stoichiometry of the Eq. 1 depends upon the atomic fraction of Fe and Al in Ca_2_(Fe, Al)_2_O_5_. In case of self-hardened pellet, the Fe_2_O_3_ in the pellet (originated from BR) is directly reduced to metallic iron via chemical reaction (3). In parallel, calcium oxide and alumina in the pellet react and yield Ca_12_Al_14_O_33_ phase. Both reactant and product phases are confirmed by XRD (Fig. 1) for both pellets. The reduction reaction of brownmillerite in sinter pellet can be written as follows:

1 $$\begin{array}{l}{7{Ca}_2\lbrack}{Fe}_x,{Al}_{1-x}\rbrack_2O_{5\left(s\right)}+21xH_{2\left(g\right)}=14xFe_{\left(s\right)}\\\;\;\;\;\;\;\;\;\;\;\;\;\;\;\;\;\;\;\;\;\;\;\;\;\;\;\;\;\;\;\;\;\;\;\;\;\;+21xH_2O_{\left(g\right)}+\left(2+12x\right)CaO_{(s)}\\\;\;\;\;\;\;\;\;\;\;\;\;\;\;\;\;\;\;\;\;\;\;\;\;\;\;\;\;\;\;\;\;\;\;\;\;\;+(1-x){Ca}_{12}{Al}_{14}O_{33(s)}\;(\mathrm s)\end{array}$$

2 $$C{a}_{2}F{e}_{2}{O}_{5(s)}+3{H}_{2(g)}\to 2F{e}_{(s)}+Ca{O}_{(s)}+{3H}_{2}{O}_{(g)},\Delta {G}_{1000^\circ{\mathrm{C}} }=\frac{27.398kcal}{mol-C{a}_{2}F{e}_{2}{O}_{5}}$$

Reduction reaction of Fe_2_O_3_ in self-hardened pellets:

3 $$F{e}_{2}{O}_{3(s)}+3{H}_{2(g)}\to F{e}_{(s)}+{3H}_{2}{O}_{(g)},\Delta {G}_{1000^\circ{\mathrm{C}} }= -\frac{36.31kcal}{mol-F{e}_{2}{O}_{3}}$$

This free energy was calculated at partial pressure p_H2/H2O_ equal to 1. However, in the actual system, the water vapor generated is much smaller than the hydrogen gas that is continuously introduced to the sample. Hence, the partial pressure p_H2/H2O_ is much higher which drives the reduction of Ca_2_Fe_2_O_5_ to right as all iron complex is reduced to metallic iron as per XRD and elemental mapping result. Here, we used Ca_2_Fe_2_O_5_ phase as a substitute of Ca_2_Al_0.204_Fe_1.796_O_5_ that we actually observed. Unfortunately, the latter phase was not found in the any thermodynamic database.

When self-hardened pellets are reduced, the mayenite (Ca_12_Al_14_O_33_) phase is formed through the interaction of CaO and Al_2_O_3_’ however, when sintered pellets are reduced, the formation of mayenite (Ca_12_Al_14_O_33_) is depending on the mass transport of oxides in the pellet matrix and more time dependent compared to the self-hardened pellets with slower rate. Due to this, the mayenite peak is more intense in reduced self-hardened pellets than the reduced sintered pellets. In the reduced sintered pellets, some of alumina is lost in the Ca_2_Al_2_SiO_7_ phase, but in the self-hardened pellets, more alumina is available for the formation of the Ca_12_Al_14_O_33_ phase. Larnite (Ca_2_SiO_4_) phase was found in both reduced samples, which is a dicalcium silicate compound, and it shows the interaction of added lime with the SiO_2_ in BR via the following reaction:

4 $$2CaO(s)+Si{O}_{2}\left(s\right)\to C{a}_{2}Si{O}_{4} (s)$$

Perovskite (CaTiO_3_) had the higher intensity in the reduced sintered pellets as compared to reduced self-hardened pellets (Fig. 2). This may be due to the high temperature sintering that leads to more interaction of added CaO and TiO_2_ in BR and formation of CaTiO_3_, which is a stable phase.

#### Mass Changes During Reduction

**Fig. 8:**
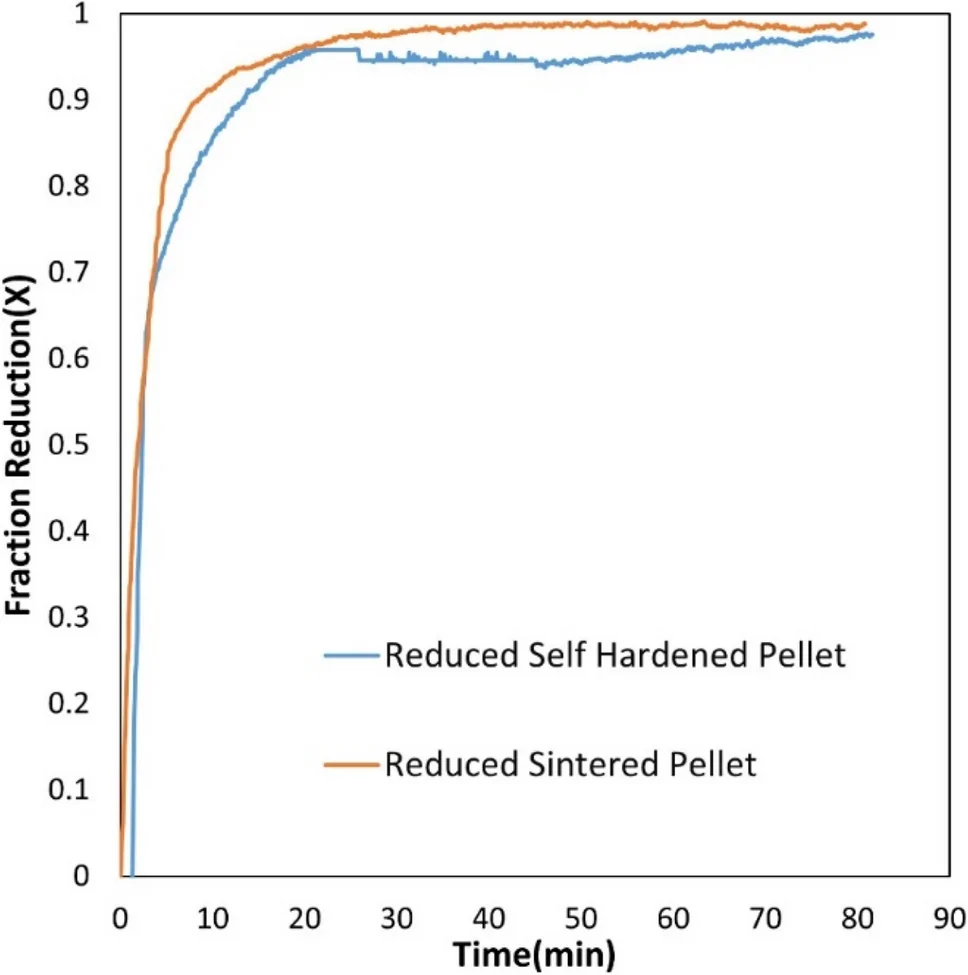
Fraction reduction of iron complex to metallic iron at 1000°C in H_2_ atmosphere

As shown in Fig. 6, the mass loss during heating cycle of the self-hardened pellets were approximately 25.2 wt%, which is due to the CaCO_3_ decomposition, aluminium hydroxide decomposition, goethite (FeO(OH)) decomposition, and chemisorbed water. As per mass balance, the CO_2_ amount from decomposition is near around 15.02 wt%. Observing the extra mass loss is due to the decomposition of alumina hydroxide compounds and goethite. There is no mass loss during heating of sintered pellets. During reduction cycle, the mass loss was about 11 wt% for both sinter reduced pellets and self-harden reduced pellets. As shown in the Fig. 8, the fraction reduction vs time, iron complex reduction to metallic iron is almost completed, which is also corelated with the theoretical mass loss and actual mass loss during reduction step. Both curves show that fraction reduction goes above 90% within a short reduction time. The mass balance of iron complex reduction to iron is shown in the Table 4. Fraction reduction is calculated based on Eq. (5):

5 $$Fraction\; Reduction\left(X\right)=\frac{ \left(\Delta {W}_{red}\right)}{ \left({W}_{in}\right)}$$

where $\Delta {W}_{red}$ is the weight loss during reduction as measured in the reduction and ${W}_{in}$ is the possible removable oxygen via the reduction.

**Table 4:** Comparison of weight loss during reduction of two different pellets

	%Fe_2_O_3_ in initial mass (g)	Theoretical mass loss (g)	Actual mass loss (g)
Self-hardened pellets	15.8	4.74	4.71
Sintered pellets	19.1	5.73	5.71

Observing close X-time behaviour during the H_2_ reduction of the pellets in Fig. 8 may indicate that the mass transport of hydrogen into the two pellets with different porosities is not a rate determining step. Moreover, the reduction of brownmillerite and hematite as the main Fe-containing compounds in the two pellets occurs with high rate. The rate of reaction of both reduced pellets is calculated as follows:

6 $$Rate\; of\; reduction =\left(\frac{Y(g)}{t(min)}\right)\left(\frac{1 mol O}{16 g O}\right)\left(\frac{1 min}{60 sec}\right)=(\frac{Y}{t})(\frac{1}{960})\left(\frac{mol}{sec}\right)$$

where *Y* is the oxygen removed during initial stage of reduction (as indicated for the curves in Fig. 6) and *t* is the time required for initial stage of reduction. By using Eq. (6), the rate of reduction of sintered reduced pellets and self-hardened reduced pellets were 1.1*10^−2^ (mol/sec) and 6.5*10^−3^ (mol/sec) respectively. The slightly different reduction rate may not be related to the pellets porosities as both have high porosities as mentioned above. However, it is worth to note that in the self-hardened pellets, we have hematite (Fig. 2), while in the sintered pellets, we have brownmillerite, and these phases may show a slightly different reduction rate.

#### Thermochemistry of the Process

**Fig. 9:**
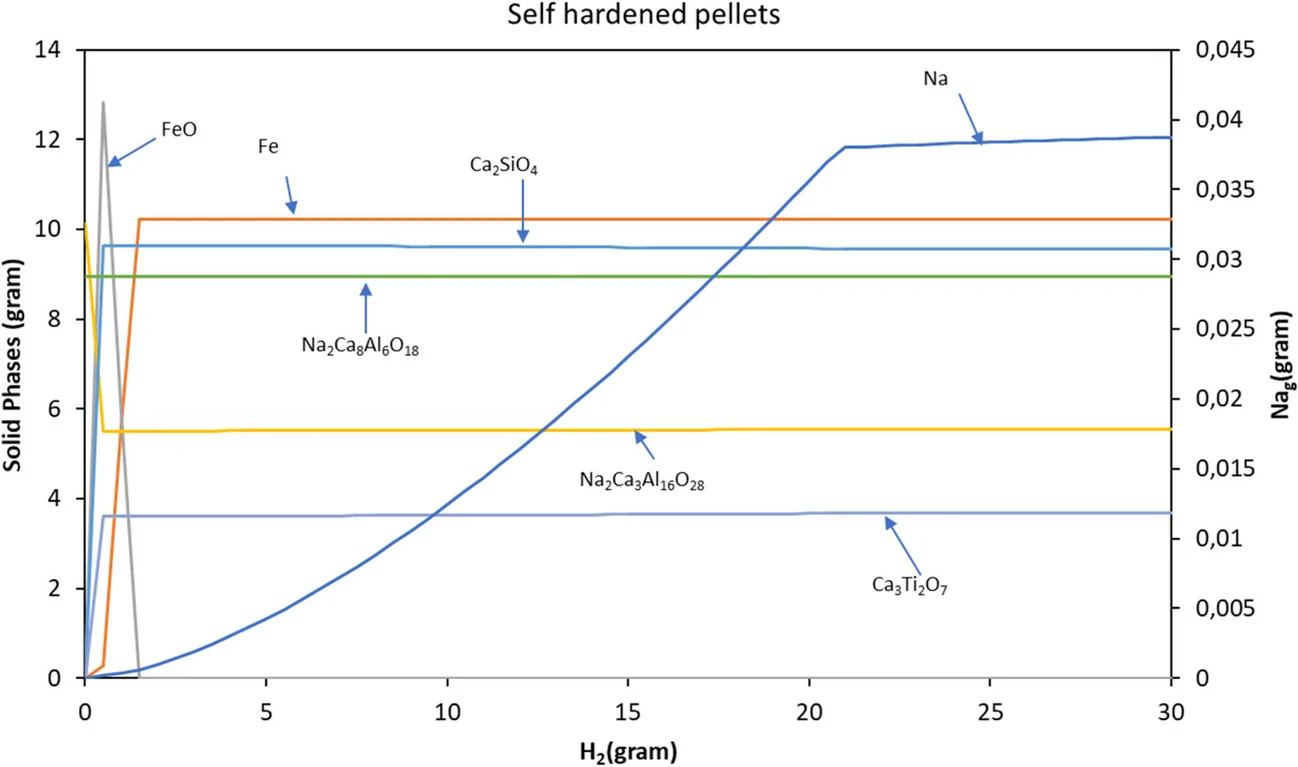
Equilibrium calculations when 50 g of self-hardened pellets reacts with H_2_ (g) at 1273 K (created using the equilibrium module in Factsage 8.1

To evaluate the obtained phases upon the reduction of both type of pellets, thermodynamic equilibrium study was carried out by using thermodynamics software (Factsage 8.1). Equilibrium was calculated based on the reduction temperature (1000 °C), reduction time (90 min), and the initial amount of different main oxide phases in the mixture. For both sinter and self-hardened pellets, the initial amount was around 50 g. The considered H_2_ gas for calculation was the total amount of gas that was used during the tests. The obtained result from the Factsage calculation was presented in Fig. 9 for self-harden reduced pellets and sinter reduced pellets respectively. Metallic iron (Fe), dicalcium silicate (Ca_2_SiO_4_), tricalcium di-titanate (Ca_3_Ti_2_O_7_), and sodium calcium aluminate (Na_2_Ca_3_Al_16_O_28_, Na_2_Ca_8_Al_6_O_18_)) were the major phases with activity one. According to XRD phase analysis of the reduced pellets, sodium calcium aluminate was not found; however, calcium aluminate phase (mayenite) was detected.

**Fig. 10:**
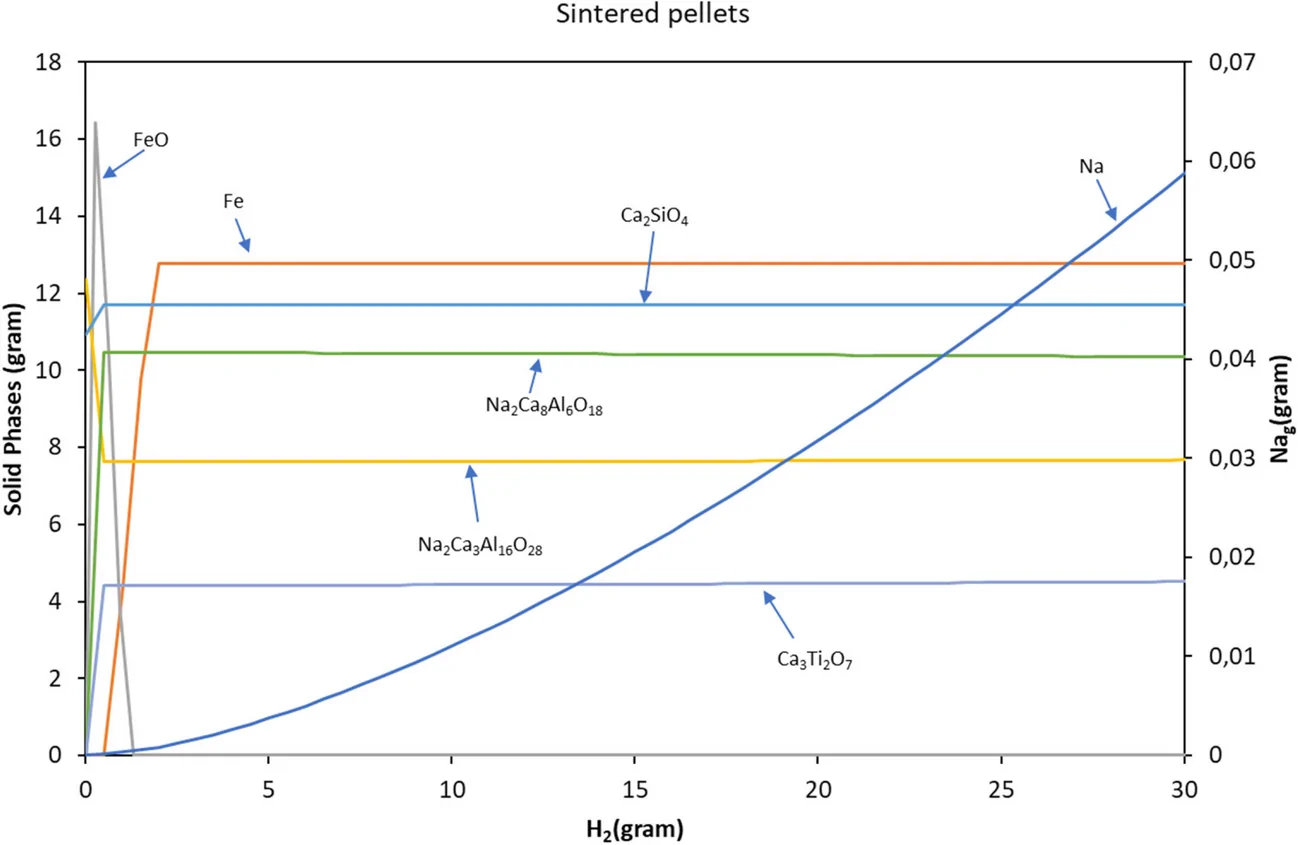
Equilibrium calculations when 50 g of sintered pellets reacts with H_2_ (g) at 1273 K (created using the equilibrium module in Factsage 8

As shown in Figs. 9 and 10, during reduction at lower amount of hydrogen, Fe_2_O_3_ is reduced to FeO; however, with increases in hydrogen amount, it reduced to metallic iron. For both the pellets with increases in hydrogen amount during reduction, more amount of Na_(g)_ is forming. For the self-hardened pellets, the formation of Na_(g)_ increases up to some amount of hydrogen; then, it becomes constant, but for sintered pellets, it increases with increase in amount of hydrogen. This may be due to the fact that in sintered pellets, the sodium present is higher as compared to self-hardened pellets. All the Fe_2_O_3_ present in the system was reduced to metallic iron from above approximately 2 g of hydrogen injected to the system.

Formation of sodium calcium aluminate in the thermodynamics calculation with respect to calcium aluminate in the actual experiment may be due to sodium losses during hydrogen reduction.

7 $$N{a}_{2}C{a}_{8}A{l}_{6}{O}_{18}+{H}_{2}=2Na\left(g\right)+C{a}_{3}A{l}_{2}{O}_{6}+2\left(CaO.A{l}_{2}{O}_{3}\right)+3CaO+{H}_{2}O$$

8 $$N{a}_{2}C{a}_{3}A{l}_{16}{O}_{28}+{H}_{2}=2Na(g)+CaO.6A{l}_{2}{O}_{3}+{H}_{2}O+2\left(CaO.A{l}_{2}{O}_{3}\right)$$

During reduction may be sodium calcium aluminate phase reduced to calcium aluminate phase and sodium metal as a vapor as per Eqs. 7 and 8.

All the thermodynamics calculation were based on consideration a closed system; however, in actual practice, we had an open system in which sodium gas loss occurs. From the EDS elemental point analysis (Fig. 5) of mayenite in both the reduced pellets, it was found that along with Ca, Al, and O, a small amount of Si and Na was present. So, from the microstructural elemental analysis, XRF analysis, and thermodynamics calculation, it may be concluded that in actual practice, formation of calcium aluminate phase with minor fraction of sodium occurs, which is not a phase in the thermodynamic database and not predicted by equilibrium calculations.

## Conclusions

Mechanical, chemical, physical, and structural properties of sintered and self-hardened BR pellets via addition of CaO and CaCO_3_ were studied in this work. Moreover, isothermal hydrogen reduction of these pellets was investigated experimentally.

It was shown that breaking load, tumbling, and abrasion indices of self-hardened pellets were lower than sintered pellets. The breaking load of the sintered pellets was 0.29 kN, which was one order higher than that of self-hardened pellets. In terms of tumbling index, the value for sintered pellets was about 88.33% which was significantly higher than that of self-hardened pellets 55.22%. Furthermore, the abrasion index of the self-hardened pellets was obtained 37.11% which was substantially greater compared to sintered pellets (4.89%).

There was negligible amount of gehlenite phase formation during reduction of self-hardened pellets compared to the sintered pellets. The intensity of metallic iron was more in reduced sintered pellets in comparison with the self-hardened ones. Reduction rate and extent reduction of iron containing oxides were very similar for both pellets. There was more formation of alumina leachable phase in reduced self-hardened pellets than the reduced sintered pellets. Further, the average iron particle size of reduced self-hardened pellets was 5–6 µm which was approximately 3–4 µm for reduced sintered pellets. Na_2_O had a dominating effect on the formation of calcium aluminate and sodium aluminate during hydrogen reduction. With an increase in Na_2_O, the formation of calcium aluminate supressed over sodium calcium aluminate.

Preliminary magnetic separation results showed the iron recovery of 41% and 27% for self-hardened and sintered reduced pellets. Moreover, low enrichment ratios were reported as 1.07 and 1.12 respectively for the self-hardened and sintered reduced pellets.

## Data Availability

The authors declare that the data supporting the fundings of this study are available within the paper.

## Appendix A

The XRD identification card for different phases of the studied BR:

1. Iron (Fe) (PDF-04–012-6482)
2. Mayenite (Al_7_Ca_6_O_16_) (PDF-04–023-4773)
3. Lime (CaO) (PDF-01–070-4068)
4. Perovskite I (CaTiO_3_) (PDF-01–089-0056)
5. Larnite (Ca_2_O_4_Si) (PDF-00–009-0351)
6. Gehlenite (Al_2_Ca_2_O_7_Si) (PDF-04–015-3030)
7. Brownmillerite (Al_0.204_Ca_2_Fe_1.796_O_5_) (PDF-04–014-6627)
8. Yeelimite (Al_6_Ca_4_O_16_S) (PDF-00–033-0254)
9. Cristobalite (SiO_2_) (PDF-04–025-9060)
10. Fayalite (Fe_2_SiO_4_) (PDF-01–071-1401)
11. Srebrodolskite (Ca_2_Fe_2_O_5_) (PDF-04–008-6821)
12. Annite (KFe_2.5_Al_2_Si_2.5_O_10_(OH)_2_) (PDF-04–017-1457)
13. Jadeite (AlNaO_6_Si_2_) (PDF-04–015-8050)
14. Calcite (CaCO_3_) (PDF-01–083-0577)
15. Hematite (Fe_2_O_3_) (PDF-01–089-0596)
16. Diáspora (AlHO_2_) (PDF-01–072-1475)
17. Cancrinite (Na_6_Al_6_Si_6_O_24_CaCO_3_(H_2_O)_2_) (PDF-01–071-0776)
18. Katoite (Ca_3_Al_2_SiO_4_(OH)_8_) (PDF-04–014-1869)
19. Perovskite II (CaTiO_3_) (PDF-00–022-0153)
20. Coesite (SiO_2_) (PDF-00–012-0711)
21. Goethite (FeOOH) (PDF-01–073-9835)
22. Sodalite (Al_6_Na_8_Cl_2_Si_6_O_24_) (PDF-01–075-0917)
23. Titanium dioxide (Ti_9_O_17_) (PDF-00–011-0431)
